# Strain-mediated ferromagnetism and low-field magnetic reversal in Co doped monolayer $WS_2$

**DOI:** 10.1038/s41598-022-06346-w

**Published:** 2022-02-16

**Authors:** Anjan Kumar Jena, Sameer Kumar Mallik, Mousam Charan Sahu, Sandhyarani Sahoo, Ajit Kumar Sahoo, Neha Kapila Sharma, J. Mohanty, Sanjeev K. Gupta, Rajeev Ahuja, Satyaprakash Sahoo

**Affiliations:** 1 Laboratory for Low Dimensional Materials, Institute of Physics, Bhubaneswar, 751005 India; 2 Homi Bhabha National Institute, Training School Complex, Anushakti Nagar, Mumbai, 400094 India; 3 Nanomagnetism and Microscopy Laboratory, Department of Physics, Indian Institute of Technology Hyderabad, Kandi, Sangareddy 502284 India; 4 Computational Materials and Nanoscience Group, Department of Physics and Electronics, St.Xavier’s College, Ahmedabad, 380009 India; 5 Condensed Matter Theory Group, Department of Physics and Astronomy, Uppsala University, 75120 Uppsala, Sweden; 6 Department of Physics, Indian Institute of Technology Ropar, Rupnagar, Punjab 140001 India

**Keywords:** Magnetic properties and materials, Information storage

## Abstract

Strain-mediated magnetism in 2D materials and dilute magnetic semiconductors hold multi-functional applications for future nano-electronics. Herein, First principles calculations are employed to study the influence of biaxial strain on the magnetic properties of Co-doped monolayer $WS_2$. The non-magnetic $WS_2$ shows ferromagnetic signature upon Co doping due to spin polarization, which is further improved at low compressive (-2 %) and tensile (+2 %) strains. From the PDOS and spin density analysis, the opposite magnetic ordering is found to be favourable under the application of compressive and tensile strains. The double exchange interaction and *p-d* hybridization mechanisms make Co-doped $WS_2$ a potential host for magnetism. More importantly, the competition between exchange and crystal field splittings, i.e. ($\Delta _{ex}>\Delta _{cfs}$), of the Co-atom play pivotal roles in deciding the values of the magnetic moments under applied strain. Micromagnetic simulation reveals, the ferromagnetic behavior calculated from DFT exhibits low-field magnetic reversal (190 Oe). Moreover, the spins of Co-doped $WS_2$ are slightly tilted from the easy axis orientations showing slanted ferromagnetic hysteresis loop. The ferromagnetic nature of Co-doped $WS_2$ suppresses beyond $\pm 2~\%$ strain, which is reflected in terms of decrease in the coercivity in the micromagnetic simulation. The understanding of low-field magnetic reversal and spin orientations in Co-doped $WS_2$ may pave the way for next-generation spintronics and straintronics applications.

## Introduction

In recent years, to develop new multi-functional materials, tremendous research efforts have been focused on two-dimensional (2D) materials due to their potential applications in areas such as electronic, opto-electronic, mechanical and chemical properties^1–3^. Among various 2D materials, transition metal di-chalcogenides (TMDCs) such as $MX_2$ (M: Mo, W, etc.; X: S, Se, etc.) marks significant attention owing to their unique potential applications in field-effect transistor (FET), photodetectors, catalysis, Li-ion batteries etc^1,2,4,5^. Ever since the discovery of graphene, weak van der Waal systems bring forth a large possibility for hosting magnetism in 2D materials. In the last few years, the focus shifted more on 2D magnetic materials, where the fundamental concept of spin dominate over charge bring new scientific properties and opens the plethora for nanoscale devices and spintronic applications, which have been demonstrated both experimentally and theoretically^6,7^. Till date several 2D material contributes distinct magnetic properties such as: (i) graphene shows excellent magnetic transport^8^, (ii) TMDCs ($MoS_2$, $WS_2$, $MoSe_2$, $WSe_2$, etc.) shows strong spin-orbit coupling (SOC) and coupled valley properties^9,10^ and (iii) 2D magnets have the potential candidate for future non-volatile memory applications^11,12^. The investigations of new 2D TMDCs or new techniques as an alternative approach have been attracted much attention towards multifunctional applications. In addition, magnetism in 2D materials is creating dilute magnetic semiconductors (DMSs), which have been extensively studied due to their charge carriers making DMSs as the potential of spintronics^13–16^.

The $MoS_2$ and $WS_2$ TMDCs show greater potential for fabricating magnetic tunnel junctions (MTJs) owing to their unique physical properties such as strong SOC, long-ranged spin diffusion length, etc^9,17^. However, these materials do not have their intrinsic magnetism and always depend on external agents. For example, a thin $MoS_2$ layer can be sandwiched between two ferromagnetic layers as a spacer to achieve MTJ properties^18,19^. Impurity doping engineering is found to be an appropriate approach to change the electronic and magnetic properties of TMDC semiconductors. Doping marks a significant change in structural stability and magnetism of graphene, which facilitates new multifunctional applications^20^. Both monolayer (ML) or stacked ($MoS_2$, $WS_2$, etc.) TMDCs semiconductors can host the magnetism after doping with either n-type or p-type impurities^21^. The Mn-doped $MoS_2$ ML depicts potential for a new class of DMSs, which is evident from first principles DFT calculations and Monte-Carlo simulations studies^22^. The ferromagnetic (FM) behavior of Mn-doped $WS_2$ has been verified experimentally and theoretically^23^. Kang *et. al.* experimentally demonstrated that the $MoS_2$ exhibits FM, while $WS_2$ shows paramagnetic behavior when doping with Fe^24^. TM-related defects complexes can induce FM in the $WS_2$ ML. Eventually, high formation energy makes it difficult for the practical growth of $WS_2$ ML. Hence, besides the doping of TM elements, vacancies or defects have also been introduced to induce magnetism in TMDC monolayers^25,26^. Furthermore, lattice strain can be adapted to modulate the physical properties of TMDCs. Tao *et. al.* predicted single-layer $MoS_2$ with single atomic vacancies shows FM under strain for the possible applications in memory switching and logic gates^27^. However, there is very few limited detailed information available on strain-induced FM in $WS_2$ systems. In addition, $WS_2$ ML has sufficiently high thermal and oxidative stability compared to $MoS_2$^28,29^. $WS_2$ ML having $P6_3/mmc$ space group symmetry, where the W atoms are having trigonal prismatic coordination with the S atoms. The presence of a covalent bond between W-S makes it suitable for magnetism in $WS_2$ after doping, which can be controlled under uniaxial/biaxial strain. Moreover, the strain-induced magnetic $WS_2$ behaves dissimilar for different types of doping. Luo *et. al.* obtained FM behavior in Al-doped $WS_2$ under applied compressive strain, while unable to produce any magnetic moment under tensile strain^30^. Contrary to this, Na-doped $WS_2$ ML shows weak magnetism at higher compressive strain, while found to be higher at greater tensile strains^31^. Therefore, these findings motivate us to study the unrevealed strain-induced magnetism in Co-doped $WS_2$ ML, which may pave the way for future spintronic applications.

In this work, we studied a possible emergence of FM in Co-doped $WS_2$ ML under strain engineering, which may have applications in TMDC based straintronics^32^. We employed DFT calculations to understand the mechanism of FM behavior in Co-doped $WS_2$ ML at biaxial compressive and tensile strain. The exact behaviour of the FM nature is understood by using crystal-field and exchange-field splitting. The system is further studied by micromagnetic simulation to address the behavior of reversal magnetization and the magnetic effects under strain. The electronic and magnetic properties are also discussed, which will be significant for future spintronic applications. More importantly, this is the first attempt to understand ferromagnetism in TMDCs of low-dimension DFT calculations with nanoscale micromagnetic simulations.

## Computational method

We perform the first principles spin-polarised DFT calculations using the Vienna ab initio simulation package (VASP)^33^ which implements the projector augmented wave (PAW) method to describe electron-ion interaction. The electronic exchange-correlation potential is described by the generalized gradient approximation (GGA) in the Perdew-Burke-Ernzerhof (PBE) parametrization^27^. The correction of a Hubbard term U (GGA+U) is necessary to describe the strongly correlated 3d orbital of the TM impurities. However, the half-metallic doped magnetic TMDC systems are found to be independent of U values (U = 2.5, 3). The value of the magnetic moments remain little affected using different U values. Additionally, the optimization of lattice constants have been performed with various U values under unstrained and strained conditions by energy minimization and are observed to be unaltered with GGA+U (see SI). Henceforth, we find no relevant modifications on our conclusions and the further discussions are based on without considering the on-site interaction. We use kinetic energy cutoff of 500$\sim$eV for the plane-wave expansion of the wave functions. All the structures are fully relaxed under conjugate gradient algorithm until a total energy convergence and Hellmann Feynman force up to $10^{-5}$ eV and 0.01 eV/ Å are achieved. The Brillouin zone integration for self-consistent and projected-density calculations is approximated by Monkhorst-Pack K point mesh of $5\times 5\times 1$ and $12\times 12\times 1$ respectively. We use $4\times 4\times 1$ and $5\times 5\times 1$ supercells for doping and strain-based calculations. The micromagnetic simulations is carried out by open freeware object-oriented-micromagnetic-framework (OOMMF)^34^. By considering the previous experimental reports^35,36^, the parameters such as magnetic anisotropy, saturation magnetization, exchange-length, etc are employed in Co-doped $WS_2$ by micromagnetic calculations.

## Results and discussion

**Figure 1:**
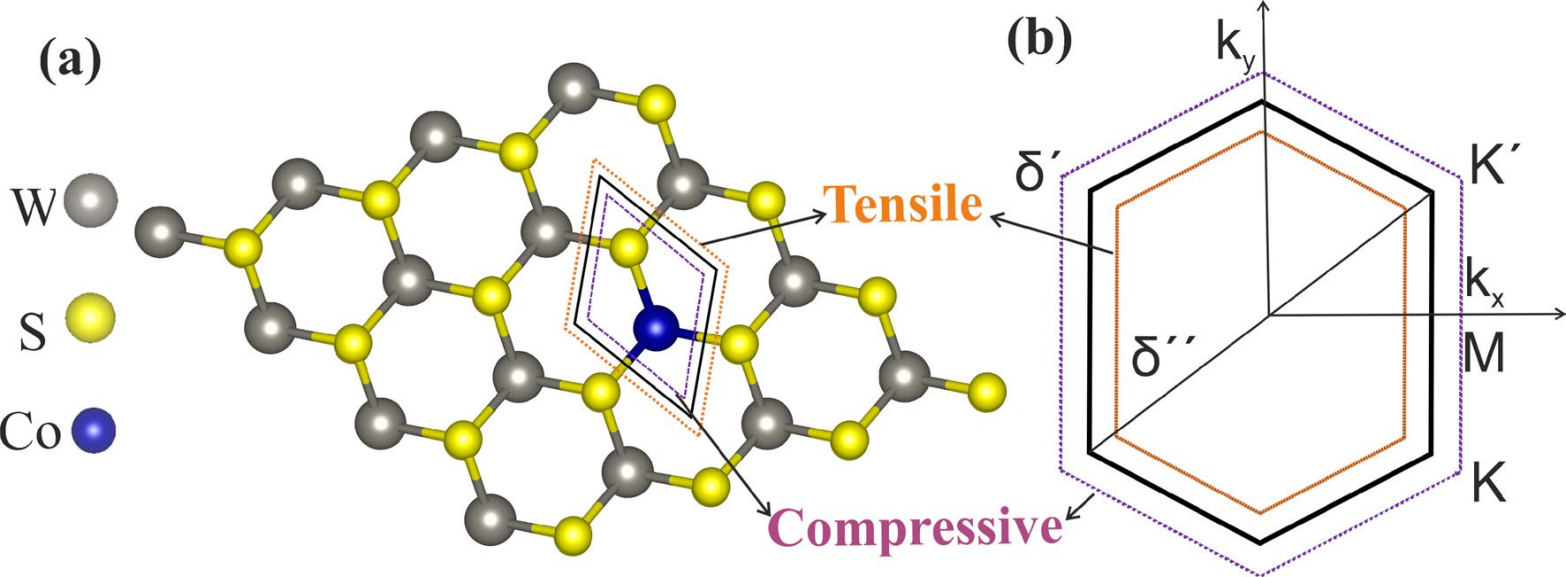
Schematic structure showing (**a**) top view of $4\times 4\times 1$ supercell Co-doped $WS_2$ monolayer. The black line represents an unstrained unit cell. The orange and violet dotted lines represents unit cells for the isotropic tensile and compressive strain, respectively. (**b**) The Brillouin Zone of $(Co, W)S_2$ ML under applied biaxial compressive (dotted violet line) and tensile strain (dotted orange line). $\delta ^{\prime }$ and $\delta ^{\prime \prime }$ are strain variation in percentage at compressive and tensile strain, respectively.

To study the effects of Co-doping for the magnetic properties of $WS_2$ mono layer (ML), we replace one host W-atom by foreign Co-atom in $4\times 4\times 1$ [$(Co, W)S_2$] and extended $5\times 5\times 1$ super-cell with doping concentrations 6.25% and 4.16% respectively. The graphical representation of $(Co, W)S_2$ ML with its hexagonal unit cell (black solid line) is shown in Fig. 1a, where the optimized lattice constant is found to be 3.16 Å. The structural and magnetic parameters extracted from the calculated results are listed in Table 1. The obtained results show that the bond lengths of W-S shrink around the doping sites locally after Co-doping when compared to pristine $WS_2$. However, the Co-S bond length remains same with W-S bond length in pristine $WS_2$ at various doping concentration. Such behavior may be attributed to various factors such as electron affinities, ionic radii differences between the Co and W ions after the geometrical relaxation and the changed electrostatic forces that arise due to the occurrence of multivalent Co ions at the host W site^37^. It is apparent to notice that $WS_2$ exhibits effective magnetic ordering with net magnetic moments $2.62~ \mu _B$ and $2.58~\mu _B$ at different Co-doping concentration of 4.16% and 6.25%, respectively. The obtained results offer similar/higher magnetic moments than the previously studied transition metal-doped $WS_2$ ML system^21^. The observation of such high magnetic moments in Co-doped $WS_2$ ML suggests its potential candidature for 2D DMSs. In order to investigate the effects of external perturbation such as strain on the magnetic properties, both compressive ($-5\%~to~ 0\%$) and tensile ($0\%~to~5\%$) isotropic biaxial strain (along x- and y-axis simultaneously) have been applied to $(Co, W)S_2$ ML. From Fig. 1a, strained unit cells (dotted orange and violet cells for tensile and compressive strain, respectively) have been shown schematically along with the unstrained unit cell. It has been previously observed that the biaxial strain attributes isotropic changes to the magnitude of the lattice vectors of various TMDCs ML^38^. The relationship between the reciprocal lattice in Brillouin zone and the primitive vectors in real space is expressed as:

1 $$\begin{aligned} a_i.b_i= 2\pi \delta _{ij} ={\left\{ \begin{array}{ll} 2\pi &{} i=j\\ 0 &{} i\ne j~(i, j=1, 2, 3.....) \end{array}\right. } \end{aligned}$$

where $a_i$ and $b_i$ are the primitive vectors and reciprocal lattice, respectively. Accordingly, under an isotropic/uniform biaxial tensile (compressive) strain, the lattice constant will be increased (decreased), and the reciprocal lattice will be shrinked (enlarged), which in turn reduces (expands) the Brillouin zone as shown in Fig. 1b^38^. During our calculations, the periodicity is well preserved under the both biaxial strains.

**Table 1:** The calculated structural parameters, and magnetic moments for undoped and Co-doped $WS_2$ ML at various Co-doping concentration.

Supercell	$d_{W-S}$ $(\AA )$	$d_{Co-S}$ (Å)	$M_{total}$ $(\mu _B)$	$M_{Co}$ $(\mu _B)$	$M_S$ $(\mu _B)$	$M_W$ $(\mu _B)$
$WS_2$	2.41	–	–	–	–	–
$(W, Co:6.25\%)S_2$	2.39	2.41	2.58	1.67	0.11	0.03
$(W, Co:4.16\%)S_2$	2.39	2.41	2.62	1.83	0.10	0.01

**Table 2:** The calculated bond lengths *Co-S* and *W-S*, bond angle *S-Co-S, magnetic moments,* and formation energy ($$E_{form}$$) of $$(Co, 
W)S_2$$ ML at different compressive and tensile strains.

Strain ($\%$)	$d_{Co-S}$ (Å)	$d_{W-S}$ (Å)	$\theta _{S-Co-S}$ (Å)	$M_T$ $(\mu _B)$	$E_{form}~(eV)$	
					W-rich	S-rich
$-5$	2.351	2.387	77.128	2.55	9.15	6.34
$-4$	2.358	2.390	78.148	2.56	7.46	4.65
$-3$	2.376	2.381	79.911	2.60	6.11	3.30
$-2$	2.381	2.384	80.914	2.69	5.09	2.28
$-1$	2.397	2.389	81.821	2.64	4.40	1.59
0	2.409	2.394	82.709	2.58	4.12	1.13
1	2.431	2.396	84.521	2.67	6.65	3.84
2	2.447	2.397	86.071	3.25	7.65	4.84
3	2.451	2.409	85.285	2.89	9.10	6.29
4	2.468	2.415	86.122	2.82	9.54	6.73
5	2.472	2.421	87.521	2.58	10.65	7.84

**Figure 2:**
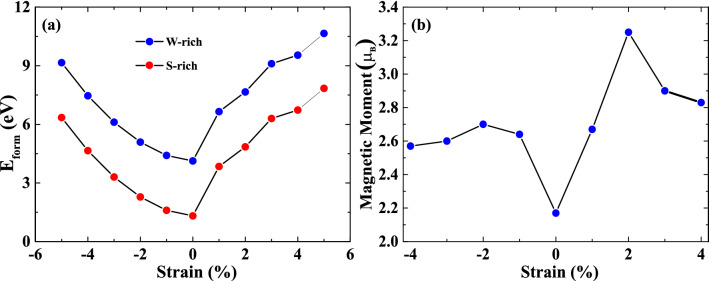
(**a**) The formation energy ($E_{form}$) of (Co,W)S_2_ ML for W-rich and S-rich conditions and (**b**) calculated magnetic moment for $(Co, W)S_2$ ML under biaxial strain, ranging from compressive − 5% to tensile +5% strains.

The structural stability of $(Co, W)S_2$ ML under isotropic biaxial strain can be estimated by formation energies, which can be expressed as: $E_{FE}= E_{doped}-E_{pure}+n(\mu _W-\mu _{Co})$, where $E_{pure}$ and $E_{doped}$ represent the total energy of the pristine $WS_2$ and Co-doped $WS_2$ ML, respectively. $\mu _W$ and $\mu _{Co}$ are the chemical potential for host W and foreign Co-atom, respectively. *n* is the number of dopants in the studied supercell. The formation energies at various biaxial strains for W- and S-rich conditions are listed in Table 2. The formation energies of the strained systems are seen to increase monotonically with the increase in both compressive and tensile strains, as displayed in Fig. 2a. Previous experimental results evident that the S-rich condition is more suitable for the practical growth of pristine $WS_2$ ML than the W-rich condition^39^. For our system, it can be noticed that $E_{form}$ for S-rich is lower when compared to W-rich conditions at each studied strain system, as supported by the experimental condition. From Fig. 2b, it can be worth noting that the magnetic moments are further modified under various applied strains, which explore possible directions for TMDCs based spintronic and straintronic application^9,32^. Among all the applied strains, the maximum magnetic moment of $3.25~\mu _B$ and $2.69~\mu _B$ are achieved for +2% tensile and -2% compressive strain, respectively. The obtained results ensure a higher magnetic moment at low strain as compared to other TM doped TMDCs^30,31,40^. According to Luo *et. al.*, the enhancement in the magnetic moments was only observed under compressive strain in Al-doped $WS_2$ ML^30^; however, the present study reveals the improvement of the magnetic moment under both compressive and tensile strain by Co-doping. The enhanced magnetic properties under strained conditions may be attributed to the overlapping of spin-polarized electronic band alignments originating from both magnetic cations and nearest neighbour anions.

**Figure 3:**
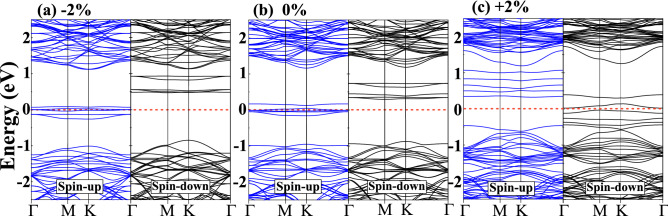
Spin-polarized band structure of (**a**) − 2% compressive, (**b**) 0% unstrained and (**c**) +2% tensile strain for single Co-atom doped $(Co, W)S_2$ monolayer. The blue and black line represents the spin-up and spin-down channels, respectively. The horizontal red dotted line indicates that the Fermi level ($E_f$) is set at zero.

The pristine $WS_2$ ML exhibits a direct bandgap of $E_g=1.87~eV$ (see SI: Fig S2) at the K-point having very close approximation with both experimental ($E_g=1.88~eV$)^41^ and other theoretical ($E_g=1.68~eV$) results^42^. Figure 3 elucidates the spin-polarized band structures of unstrained and strained $(Co,W)S_2$ ML at their equilibrium lattice constant. However, it can be noticed from Fig. 3b that some impurity states appear within the bandgap when a single W-atom is replaced by Co in both spin-up and spin-down channels. These impurity states are mainly contributed by Co-atom, while the effects from the nearest neighboring W and S atoms can be neglected, which has been explained in other TM doped TMDCs^21^. The asymmetric behavior of spin-up and spin-down components of Co-doped $WS_2$ ML indicates $WS_2$ behaves magnetically active after Co-doping. Moreover, the doped ML systems show half-metallic characteristics due to the suppression of bandgap. The spin-up component in the doped band structure shows dispersion behavior due to crossing of band lines across the Fermi level, as shown in Fig. 3b. Similarly, the spin-down component of the doped system has a bandgap of 1.168 eV with a half-metallic spacing of 0.294 eV for $(Co, W)S_2$ ML. Furthermore, the tuning of bandgap at various isotropic biaxial strains brings out effective variations in the magnetic properties of the doped system. The increase in the bond-lengths and bond-angles under various isotropic tensile strains are listed in Table 2. This indicates the decrease in the bond-energy between TM elements and S atoms leads to increase the number of spin-polarized electrons at the vicinity of fermi-level. As a result of which, the maximum obtained magnetic moment ($3.25~\mu _B$) is observed at +2% tensile strain. Figure 3a and c shows the spin-polarized band structure under the application of −2% compressive and +2% tensile strain, respectively. Moreover, one can infer that in the case of −2%, spin-up impurity states are localized near the Fermi level occupying Co-d states, whereas, for +2%, the spin-down Co-d states are filled to produce the magnetism. This behavior can be correlated to the opposite nature of the magnetic ordering in compressive and tensile strains and may show good performance in transferring one particular spin oriented electrons such as in a spin filtering devices. The highly dispersive impurity d-band lines around the fermi level in case of +2% tensile strain renders more electrons on the edge of Co atom spin-polarised when compared to the less dispersive d-band lines in case of −2% compressive strain. Interestingly, the bandgap is minimum at strain $\pm 2\%$, which ensures the bandgap tunability plays a significant role in TMDC based DMSs. The variation of half-metallic gaps with two different U values (U = 2.5, 3) under unstrained and strained (both compressive and tensile) conditions have also been incorporated which are listed in supplementary information (see SI: Table S3). However, the bandgap is further increased at higher applied strains (see SI: Fig. S2) resulting in the decrease of the magnetic moments in both strain direction.

**Figure 4:**
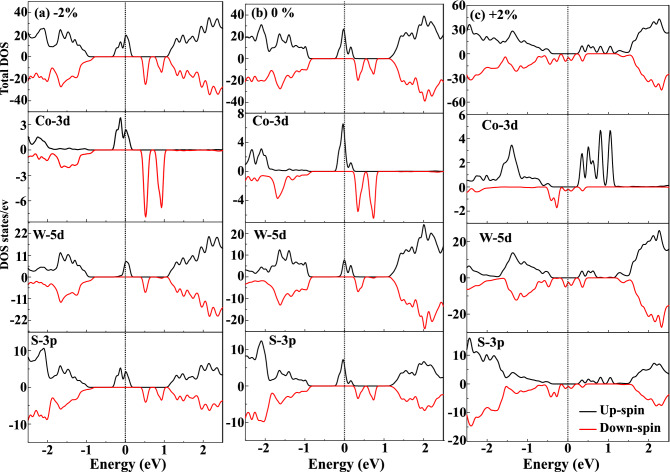
The total density of states (TDOS) and partial density of states (PDOS) of (**a**) − 2% compressive, (**b**) 0% unstrained and (**c**) +2% tensile strain for $4\times 4\times 1$ supercell $(Co, W)S_2$ monolayer. The horizontal line indicates that the Fermi level ($E_f$) is set at zero.

To further elucidate the electronic and magnetic properties in strained $(Co, W)S_2$ ML, the total density of states (TDOS) and partial density of states (PDOS) are plotted in Fig. 4. The pristine $WS_2$ behaves as a non-magnetic semiconductor, which can be inferred from the symmetric nature of spin-up and spin-down states (see SI: Fig. S3), which is well consistent with previously studied results^42^. Unlike pristine $WS_2$, in $(Co, W)S_2$ ML, the splitting of spin states is observed, giving rise to net magnetic moment ($2.58~\mu _B$), as depicted in Fig. 4b. The origin of magnetization in $(Co, W)S_2$ ML is mainly contributed from the additional three unpaired electrons of $Co-3d^74S^2$ than $W-5d^{4}4s^2$ with $1.67~\mu _B$ per Co-atom giving rise to n-type doping. However, the contribution from the nearest neighbor W ($0.03~\mu _B$ per W atom) and S ($0.11~\mu _B$ per S atom) atoms to the net magnetic moment is less than the contribution arising from Co. Similarly, the contribution from the Co-atom and the nearest neighbour W, S atoms at various studied compressive and tensile strains are listed (see SI: Table S1). The TDOS and PDOS for extended $5\times 5$ supercell (see SI: Fig. S3) show no significant modification in magnetization after Co-doping. To understand the magnetic exchange behavior in $(Co, W)S_2$ ML, the interaction between foreign Co-*d* with neighbouring W-*d* and S-*p* are need to be considered. The values of magnetic moments depend on the hybridization among the Co-*d*, W-*d* and S-*p*. The occupied states near the Fermi level mainly arise from Co-3d and W-5d orbitals in the majority spin channel, as evident from Fig. 4b. As the Fermi level lies within the partially occupied majority band of the impurity states, expecting a double exchange coupling between Co and W^43,44^. Similarly, from Fig. 4a, it can be inferred that the broadening of the majority bands along with the Fermi level passing through them indicates a stronger double exchange mechanism under compressive strain^40^. However, in tensile strain, the broadening of the minority charge carriers can be noticed across the Fermi level forming highly delocalized minority spin channels which allows maximum magnetic moment under strained $(Co, W)S_2$ ML. The interaction of Co-*d* with nearest adjacent S-*p* can be explained in terms of *p-d* hybridization mechanism. The Co atoms are strongly coupled with their neighboring S atoms around the doping sites due to the hybridization of out-of-plane Co-*3d* and S-*3p* orbitals creating considerable unbalanced spin populations in spin-split impurity bands near the fermi level. It is worth noting from Table 2 that the Co-S bond length is lower than the W-S bond under compressive strain, which is found to be reversed in the case of applied tensile strain. This change in bond lengths leads to different hybridization mechanisms for varied strains. From the above analysis, it can be believed that the competition between the Co-S and W-S bond lengths may be considered as the prime factor in modulating the magnetic properties under strain engineering.

**Figure 5:**
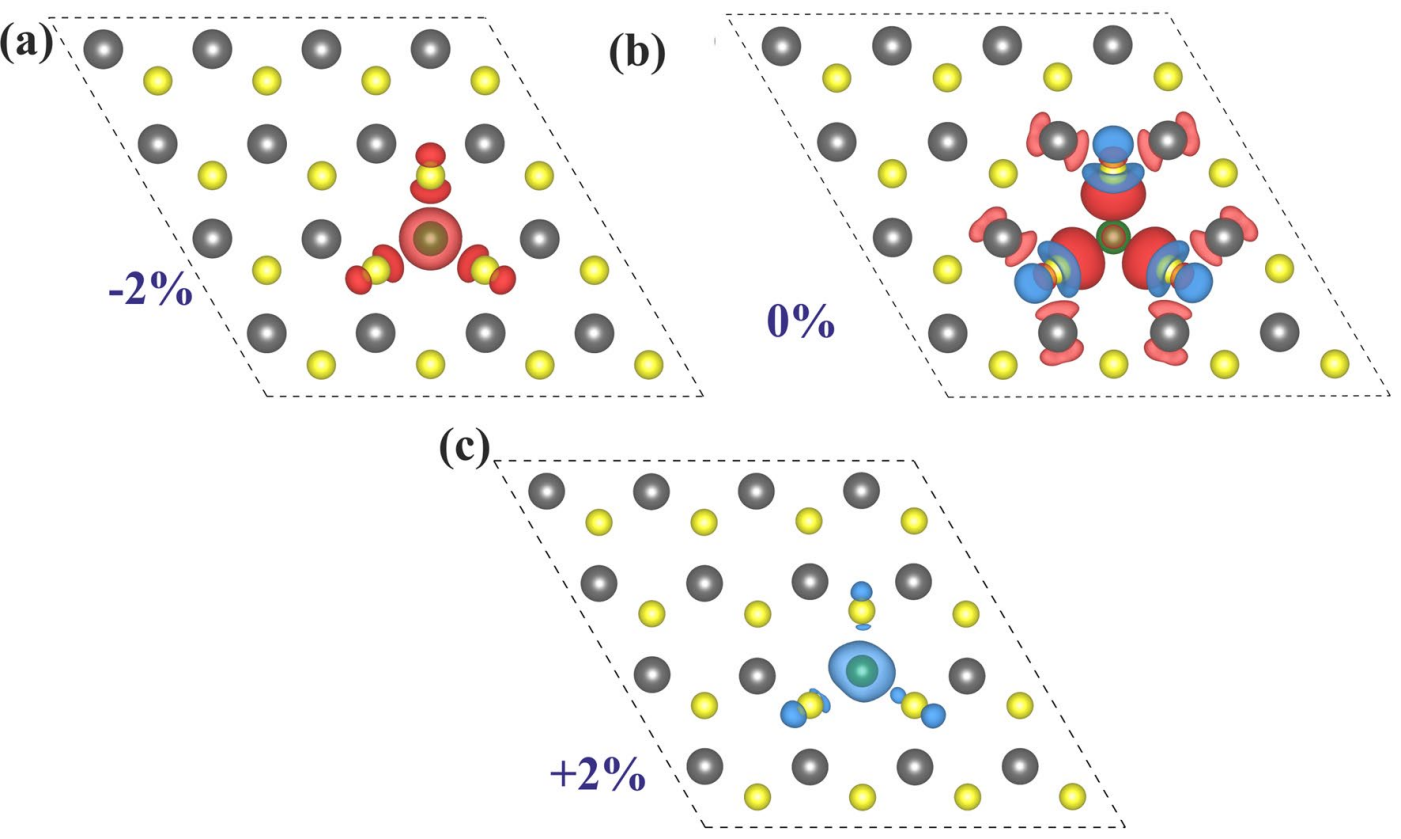
Spin density for a single Co-atom doped $(Co, W)S_2)$ ML at (**a**) − 2% compressive, (**b**) unstrained and (**c**) +2%. Red and blue isosurfaces represent positive and negative spin densities ($\pm 0.008$ e/Å^3^), respectively.

Next, we consider the spin density distribution to support our understanding of the exchange coupling from the PDOS near the Fermi level, shown in Fig. 5. Figure 5b elucidates the spin polarization between the Co atom and its nearest W/S in unstrained $(Co, W)S_2$ ML. The coupling between the foreign Co-atom and host W-atom results in parallel spin alignment indicating a double exchange interaction as evident from PDOS in Fig. 4b. However, the interaction between Co and nearest neighbor S results in *p-d* hybridization from the out-of-plane orbitals as depicted from Fig. 5b. The magnetic coupling behavior in a TM doped TMDCs systems can also be tuned by the introduction of cation and anion vacancies^45^. In such cases, the trapped electrons in the vacancies couple with the magnetic moments of the TM impurity ions form bound magnetic polarons (BMP) within the Bohr radius of the impurity site. The recently studied experimental results confirms the strong ferromagnetism induced by BMP driven by S-vacancies in $MoS_2$ nanosheet^46^. However, in our DFT calculations, the magnetic properties of Co-doped $WS_2$ monolayer have been established by considering the S-rich environment due to their lower formation energies. Additionally, The contribution from BMP and S-deficiency-induced spin delocalization in Co-doped $WS_2$ monolayer under both unstrained and strained condition has weak effect to the ferromagnetism. From the spin density distribution of $(Co, W)S_2$ ML, it can be perceived that Co-doping induces a long-range magnetic interaction with nearest neighboring W/S atoms. Moreover, the spin distribution is more localized around its magnetic centres for strained systems when compared the spin distribution around the magnetic centres of unstrained system. In case of unstrained $(Co, W)S_2$ ML, a continuous network of the magnetically coupled TM impurities over local clusters establishes a long-range magnetic interaction leading to lower percolation threshold. However, under the application of compressive and tensile strains, the local spin clusters around the impurity atoms establishes a short-range magnetic interaction, which limits to the nearest neighbour S atoms. In such a scenario, a higher doping concentration is required to reach the percolation threshold^47^. Additionally, it can be concluded that the induced spin density at the dopant site is maximum under +2% tensile strain, which reflects the ultimate magnetic moment in this case. For the extended $5\times 5\times 1$ Co-doped $WS_2$ supercell, the magnetic coupling between dopant and host atoms shows similar behavior (see SI: Fig. S4). However, with the increase in either compressive or tensile strain, the magnetic moment decreases due to lower spin polarization.

**Figure 6:**
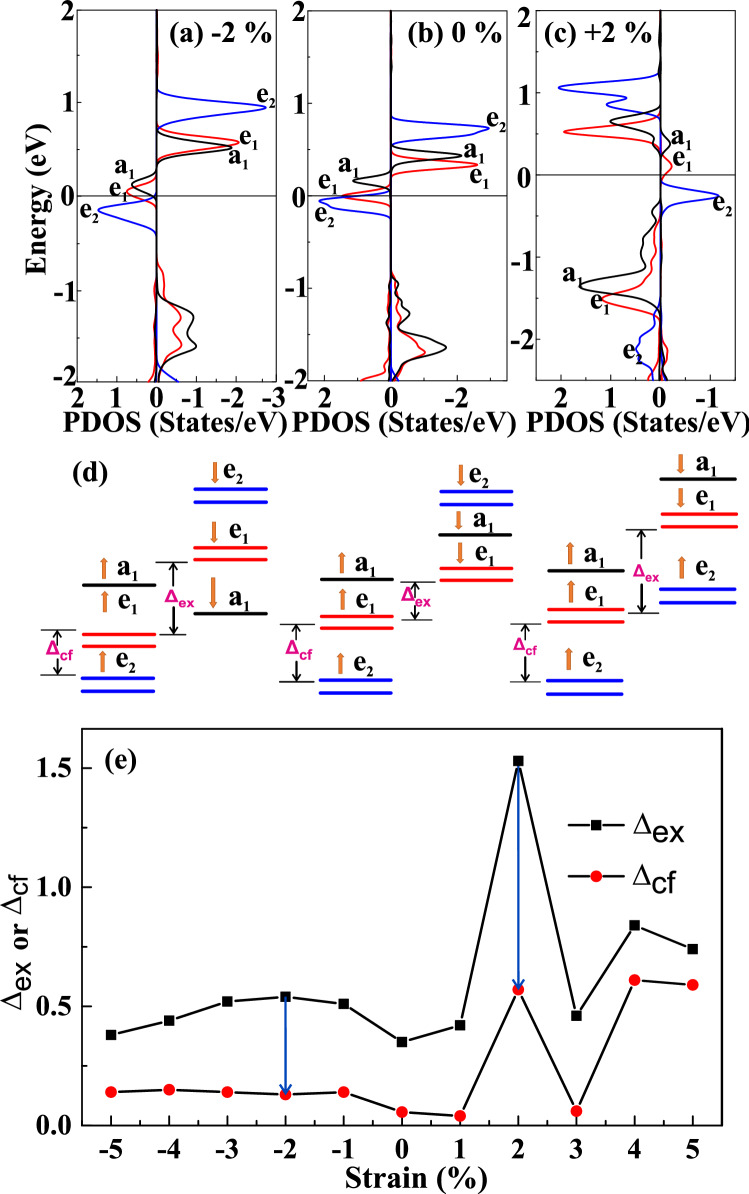
Orbital decomposed partial density of states (PDOS) of Co-doped $WS_2$ ML at (**a**) − 2%, (**b**) 0% and (**c**) +2%. (**d**) d-orbital splitting of Co-dopant at various applied strain. $\Delta _{ex}$ and $\Delta _{cf}$ represents the intra-atomic Hund’s exchange splitting and crystal field splitting, respectively. (**e**) The exchange splitting ($\Delta _{ex}$) and crystal filled splitting ($\Delta _{cf}$) at various applied compressive and tensile strains. The vertical arrow indicates the separation between $\Delta _{ex}$ and $\Delta _{cf}$.

**Figure 7:**
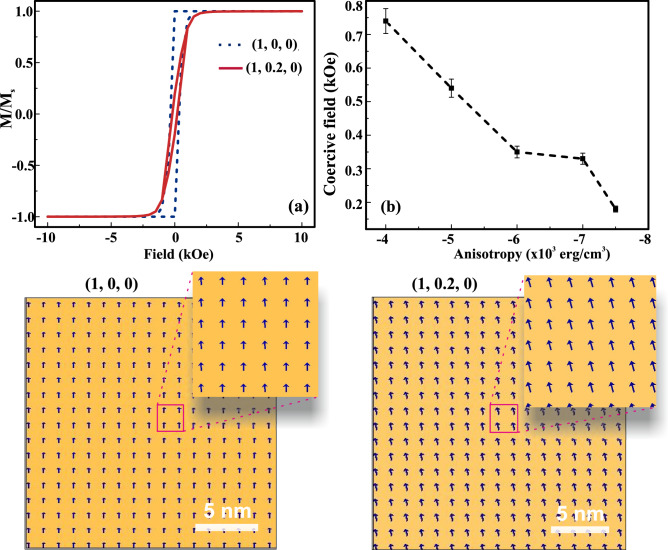
Simulated hysteresis and magnetic domains: (**a**) represents two loops obtained for (1, 0, 0), and (1, 0.2, 0) orientation. The ground state corresponding domains are marked bottom of it. (**b**) The variation of coercive field with the anisotropy values represents the strain induced ferromagnetism in Co-doped $WS_2$ monolayer.

The origin of FM behavior in $WS_2$ ML after doping and strain engineering can be further explained based on orbital decomposed PDOS analysis of the Co-atom, as shown in Fig. 6. According to ligand field theory, the 3d states of Co atom can be split into single [$a_1$ ($d_{Z^2}$)] and two two-fold degenerate [$e_1~(d_{xy, x^2-y^2})$, $e_2~(d_{xz,yz}$] states. Intra-atomic Hund’s exchange splitting ($\Delta _{ex}$) is determined by the energy difference of $e_1$ orbital between the spin-up and spin-down states, whereas the energy difference between $e_1$ and $e_2$ orbitals is referred to as crystal field splitting ($\Delta _{cf}$)^48,49^. The spin-splitting in Co-doped and strain engineered $WS_2$ near the Fermi level mainly results from the exchange splitting and crystal field splitting. As suggested by Pan *et. al.*, the FM behavior in TMDCs due to TM doping arises from the competition between the exchange splitting and crystal field splitting^50^. Figure 6d represents the schematic for exchange and crystal field splitting under − 2% compressive and +2% tensile strain compared with the unstrained condition. Moreover, the exchange splitting dominates over crystal field splitting in our studied system due to n-type Co-dopant. From Fig. 6d, it can be observed that the difference between $\Delta _{cf}$ and $\Delta _{ex}$ increases under the application of strains, which in turn reflects the increased magnetic moments, as listed from Table 2. This $\Delta _{ex}$ and $\Delta _{cf}$ at each studied strain is plotted in Fig. 6e. The detailed analysis confirms that the larger the separation between $\Delta _{cf}$ and $\Delta _{ex}$, the greater will be magnetic moments, as evident from Fig. 6e^50^.

Our detailed observation from the DFT calculation reveals that the Co-doped $WS_2$ ML behaves as FM in nature. In order to better implement an application point of view, the magnetization reversal of $WS_2$ under Co-doping engineering needs to be understood. After substituting Co at W-site, there are two possibilities of formation of anisotropy, one is uniaxial, and another one is biaxial anisotropy. As suggested from the previous reports, the uniaxial anisotropy value is high due to the formation of a larger coercive field ($H_c$) with the easy axis measurement ^51^. In contrast, the biaxial anisotropy strength is lower, resulting in low $H_c$ values. Experimental results show that the Co-doped $WS_2$ bulk, nanosheets, ML exhibit hysteresis at very low $H_c$ (few hundreds of Oe) at room temperature^35,36,52^. A similar FM signature is also reported in the Co-doped $WSe_2$ system^36^. However, the nature of the hysteresis is found to be slanted in all these cases. This intrigues us to understand the behavior of magnetization in our system. However, the FM behavior in Co-doped in $WS_2$ has not been thoroughly understood in terms of magnetization reversals. The quest for slanted hysteresis in all the reported cases is still elusive in the scientific community. Here we address the behavior of magnetization reversal and the magnetic effects of the system under strain. We approach micromagnetic modeling by using an open freeware object-oriented-micromagnetic-framework (OOMMF) package to perform qualitative analysis for ferromagnetism^34^. This is a to understand the intrinsic magnetic properties of the FM systems, which has very limited reports available on TMDCs materials. For practical applications, we need a sample dimension of nm-in-range. For micromagnetic modeling, we used a sample dimension of $50\times 50\times 1~nm^3$, and cell size is $1\times 1\times 0.5~nm^3$ to compute the simulation. This micromagnetic simulation governs by Landau-Lifshitz-Gilbert (LLG) equation, can be written as^53^:

2 $$\begin{aligned} \frac{d\mathbf{M} }{dt}=-\frac{\gamma }{1+{\alpha }^2}\mathbf{M} \times \mathbf{H} _{eff}-\frac{\gamma \alpha }{(1+{\alpha }^2) M_s} \mathbf{M} \times (\mathbf{M} \times \mathbf{H} _{eff}) \end{aligned}$$

$\frac{d\mathbf{M} }{dt}$ provides the information of the **M** over time, first term represents precision of moments, while the second term is responsible for damping. $\gamma$ denotes the gyromagnetic ratio, $\alpha$ stands for damping factor, these values are kept constant throughout the process of simulation. $M_s$ represents saturation magnetization, and $\mathbf{H} _{eff}$ is the effective field of demagnetization and external magnetic field. $M_s$ is correlated with the total energy and $\mathbf{H} _{eff}$ of the system, $\mathbf{H} _{eff}= -(E_{total}/\mathbf{M} )/(\mu _0 M_{s}$), where $E_{total}$ is the total energy density of the system. $E_{total}$ would be the sum of all micromagnetics energies, which can be written as:

3 $$\begin{aligned} E_{total}= E_{exch} + E_{anis} + E_{demag} + E_{Zeeman} + E_{me} \end{aligned}$$

$E_{exch}$ is the exchange energy, $E_{anis}$ is the magnetocrystalline anisotropy energy, $E_{demag}$ is the demagnetizing or stray field energy, $E_{Zeeman}$ is due to an external field, and $E_{me}$ is the magnetoelastic energy.

In the micromagnetic simulation, magnetic parameters are set the same throughout the cells. A stable state is achieved from a random energetic state. The simulation is performed implementing finite difference methods. The parameters are optimized by looking at various Co-doped systems and the magnetic properties associated with atomic layers of Co films^51^. The optimized parameters like exchange length: $2.1\times 10^{-6}~erg/cm$, saturation magnetization: $30~emu.cm^{-3}$, anisotropy values: $(4-6)\times 10^{-3}~ erg/cm^3$ are considered for micromagnetics simulation. When Co is doped initially at W-site, the magnetic moments are randomly dispersed in the cell matrix. On applying a sufficient magnetic field, the Co moments get oriented in the direction of external field. The snapshots of the various simulated states for the orientation of the spin moments while achieving from a random state to uniform ground state with systematic increase in applied magnetic field is shown in the supplementary information (see SI: Fig. S5). A single magnetic domain state is observed throughout the hysteresis. Figure 7a represents two kinds of hysteresis taken at two different easy axes. The hysteresis for (1, 0, 0) is quite square-in-nature, where the nucleation of domains occurred near to the remanence. However, this signature of the loop contradicts the loop obtained for Co-doped in $WSe_2$, and $WS_2$ ML systems^35,36^. To achieve the experimental signature of the loop, which is slightly slanted and nucleation occurs at a distance from the remanence, we consider magnetization orientation tilted by 20% off from the original orientation, i.e, (1, 0.2, 0) direction. As a result, the obtained hysteresis is slightly slanted from the previous orientation (1, 0, 0), which is nearly similar to the hysteresis obtained in the $WS_2$ system^35^. From the previously studied experimental results, it has been observed that $WSe_2$ shows relatively strong ferromagnetism after doping with TM Co^36^. The experimental observation shows tilting magnetization hysteresis curve at all studied temperatures ranging from very low 5 K to 300 K^36^. A similar FM response was reported in a wide range of temperature including room temperature of $WS_2$^35^. Although the magnetic parameters of $WS_2$ system are tuned for various factors such as size dependent (power and nanoribbons), thermal effect, doping, defect engineering, etc.; but, the tilting behavior is persist with all conditions^25,35,36,52,54^. Hence, the behavior of tilting M-H behavior has unaffected under these conditions, which ensures the anisotropic energy of the system attributes the tilting behavior in magnetization hysteresis curve of TMDCs. The coercive field for (1, 0, 0) orientation is around 345 Oe, whereas the same has been reduced to 190 Oe for (1, 0.2, 0). This coercive field is quite agreeing with the result reported in the $WS_2$ system^35^. Therefore, after Co doping into the system, it is worthy of mentioning that the effective magnetization orientation is slightly off from the easy anisotropic axis.

From DFT calculations it is evident that the FM behavior can be tuned under the application of strain. Here, the micromagnetic simulation provides the effect of strain on the larger scale in Co-doped $WS_2$ ML. In this case, if strain is applied to the system, we expect an alteration in the anisotropic values. In the literature also, it is reported that strain can certainly control the anisotropy in thin films^55,56^. Here, we vary anisotropy values to understand the effect of strain in the Co-doped $WS_2$ system. In this case, we tune the anisotropy values from $-4\times 10^{-3}~erg/cm^3$ to $-7.5\times 10^{-3}~erg/cm^3$ to observe the changes in the magnetic properties. The coercive field is gradually decreased with the increase in the anisotropy value, which is represented in Fig. 7b. On the other way, FM properties are getting affected by enhancing anisotropy values. This behavior is quite analogous with the results obtained from DFT calculations, where the increase in the percentages in strain values leads to a decrease in FM nature. Our results reinvigorate the FM coupling behavior in Co-doped $WS_2$ ML under various strain conditions; a further understanding of magnetic reversal in this system may pave the way for next-generation spintronics and straintronics applications.

## Conclusion

We explore the strain-induced ferromagnetism in transition metal Co-doped $WS_2$ monolayer by using first-principles DFT calculations and micromagnetic simulation. Co-doping marks a significant change in magnetic properties with an impressive magnetic moment of $2.58~\mu _B$. The magnetic exchange interaction is found to be double exchange coupling between Co and W and strong *p-d* hybridization between Co and nearest S, which is further verified from spin density distribution. We find that the resultant impurity bands of the Co-doped $WS_2$ plays a role of seed to drive novel electronic and magnetic properties under applied strain. Among several biaxial strains, the magnetic moment is found to be a maximum of $3.25~\mu _B$ at 2% tensile and $2.69~\mu _B$ at −2% compressive strain due to strong double exchange coupling and *p-d* hybridization among foreign Co and W/S. Further magnetic moments at higher applied strain is decreased due to reduced spin polarization. In addition, the competition between exchange splitting and crystal field splitting of Co *d*-orbital plays a significant role to determine these values of magnetic moments under the application of strain. From the micromagnetic simulation, it is confirmed that the Co-doped $WS_2$ monolayer shows slanted ferromagnetic hysteresis with a low coercive field. The effect of higher strain suppresses the ferromagnetic nature, which has a good agreement with the results obtained from DFT calculations. Our findings indicate that induced magnetism in $WS_2$ monolayer under Co-doping promotes the application of 2D TMDCs for the nano-scale spintronics, and especially, the strain-mediated magnetism can be a promising candidate for future straintronics applications.

## Supplementary Information

Supplementary Information.
