# Supplementary material for: Strain-mediated ferromagnetism and low-field magnetic reversal in Co doped monolayer WS2
Source: Sci Rep. 2022 Feb 16;12:2593. doi: 10.1038/s41598-022-06346-w (PMC8850603; doi:10.1038/s41598-022-06346-w)
Supplement: Supplementary file 1 — Supplementary Information. [file 41598_2022_6346_MOESM1_ESM.pdf]

Strain-mediated Ferromagnetism and Low-field Magnetic Reversal in Co doped monolayer WS_2

Anjan Kumar Jena[1,2,+], **Sameer Kumar Mallik**[1,2,+], **Mousam Charan Sahu**[1,2], **Sandhyarani Sahoo**[1,2], **Ajit Kumar Sahoo**[3], **Neha Kapila Sharma**[1,2], **J. Mohanty**[3], **Sanjeev K. Gupta**[4,*], **Rajeev Ahuja**[5,6], **and Satyaprakash Sahoo**[1,2,*]

[1]Laboratory for Low Dimensional Materials, Institute of Physics, Bhubaneswar 751005, India
[2]Homi Bhabha National Institute, Training School Complex, Anushakti Nagar, Mumbai 400094, India
[3]Nanomagnetism and Microscopy Laboratory, Department of Physics, Indian Institute of Technology Hyderabad, Kandi, Sangareddy 502284, India
[4]Computational Materials and Nanoscience Group, Department of Physics and Electronics, St.Xavier's College, Ahmedabad 380009, India
[5]Condensed Matter Theory group, Department of Physics and Astronomy, Uppsala University, S-75120 Uppsala, Sweden
[6]Department of Physics, Indian Institute of Technology Ropar, Rupnagar, Punjab-140001, India
[*]sanjeev.gupta@sxca.edu.in and sahoo@iopb.res.in
[+]these authors contributed equally to this work

Table S1. The total magnetic moment and contribution from the foreign Co dopant and nearest neighbour S and W atoms in the $(Co,W)S_2$ supercell at various compressive and tensile strains.

Strain (%)	M_T (μ_B)	M_{Co} (μ_B) per Co atom	M_S (μ_B) per S atom	M_W (μ_B) per W atom
-5	2.55	1.67	0.10	0.03
-4	2.56	1.71	0.11	0.03
-3	2.60	1.79	0.11	0.02
-2	2.69	1.84	0.11	0.02
-1	2.64	1.89	0.11	0.02
0	2.16	1.67	0.11	0.03
1	2.67	1.52	0.02	0.04
2	3.25	2.59	0.04	0.08
3	2.89	1.02	0.14	0.05
4	2.82	2.17	0.11	0.02
5	2.58	2.16	0.13	0.01

Table S2. The table comparing the total magnetic moment calculated from GGA and GGA+U methods at various strained (-2%, 0%, and +2%) conditions for $(Co,W)S_2$ ML.

Strain		-2 (%)			0 (%)			+2 (%)	
	U=0	U=2.5	U=3	U=0	U=2.5	U=3	U=0	U=2.5	U=3
Moments (μ_B)	2.69	2.73	2.75	2.58	2.69	2.78	3.25	3.27	3.28

Table S3. A table comparing the tuning of bandgap (E_g) calculated from GGA and GGA+U methods at various strained (-2%, 0%, and +2%) conditions for $(Co,W)S_2$ ML.

Strain		-2 (%)			0 (%)			+2 (%)	
	U=0	U=2.5	U=3	U=0	U=2.5	U=3	U=0	U=2.5	U=3
Bandgap (E_g)	0.11	0.13	0.12	0.16	0.17	0.13	0.09	0.10	0.12

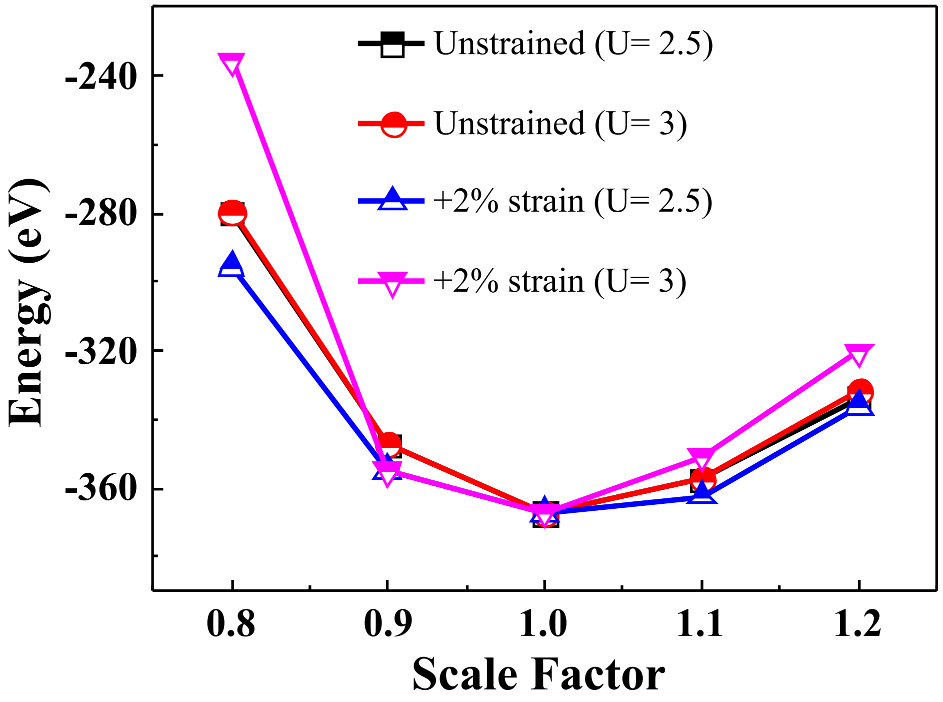

Figure S1. The variation of energies with different scale factors at U= 2.5 and U= 3 for unstrained and strained (+2%) $(Co,W)S_2$ ML.

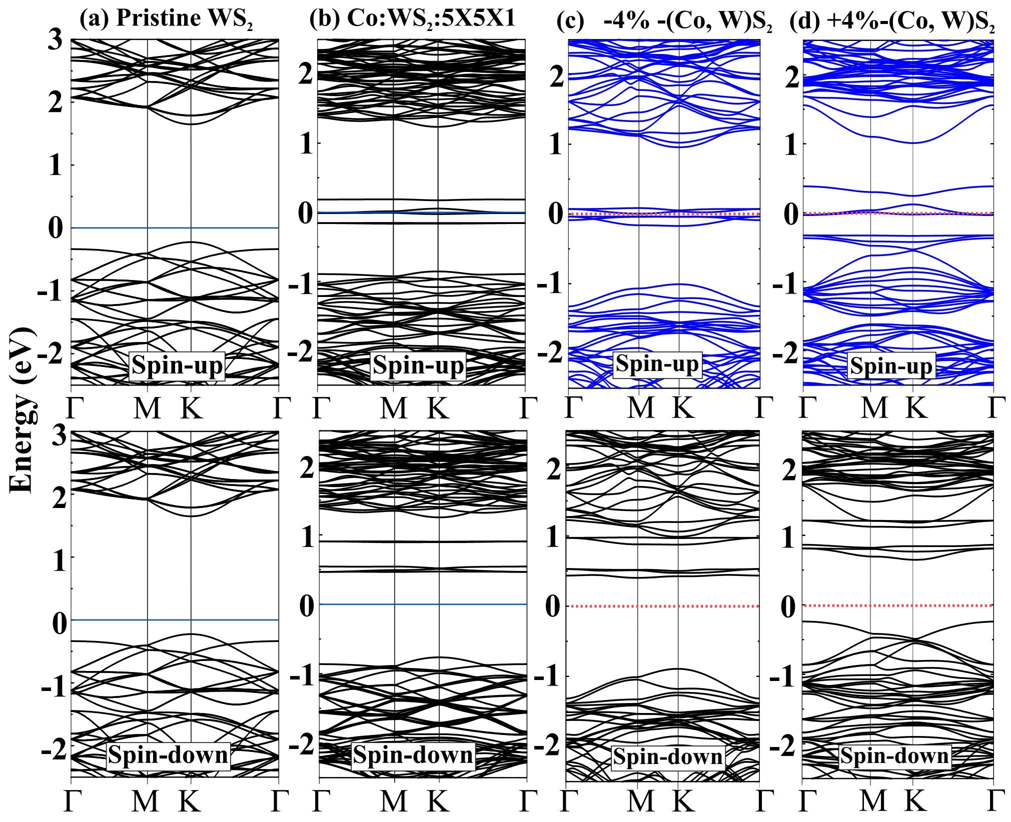

Figure S2. Spin-polarized band structure of (a) pristine WS_2, (b) single Co-atom doped WS_2 monolayer, $5 \times 5 \times 1$ supercell (c) -4% compressive strain, and (d) +4% tensile strain. The horizontal red dotted line indicates that the Fermi level (E_f) is set at zero.

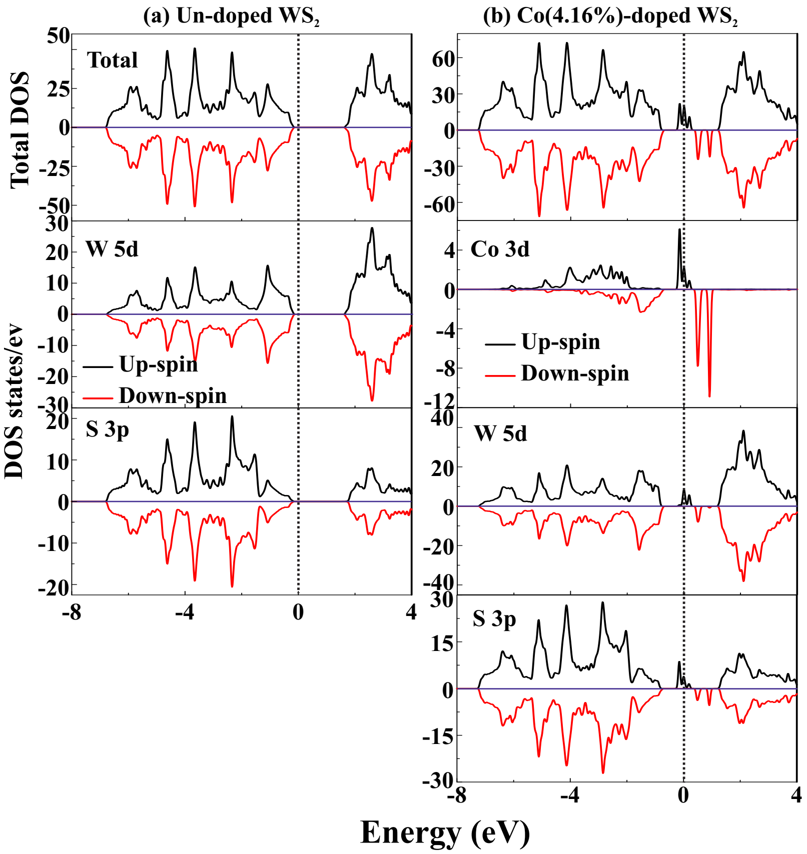

Figure S3. The total density of states (TDOS) and partial density of states (PDOS) of $4 \times 4 \times 1$ supercell (a) Pristine and (b) Co-doped WS_2 monolayer ($5 \times 5 \times 1$ supercell). The upper part and lower part of the band structure represents the spin-up and spin-down channels. The horizontal blue line indicates that the Fermi level (E_f) is set at zero.

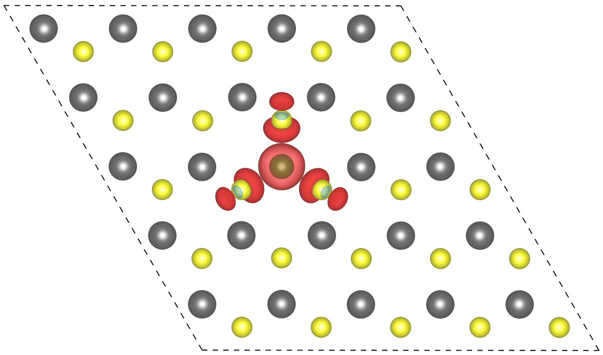

Figure S4. Spin density for a single Co-atom doped WS_2 monolayer for extended $5 \times 5 \times 1$ supercell. Red and blue isosurfaces represent positive and negative spin densities ($\pm 0.008 e/AA^3$), respectively.

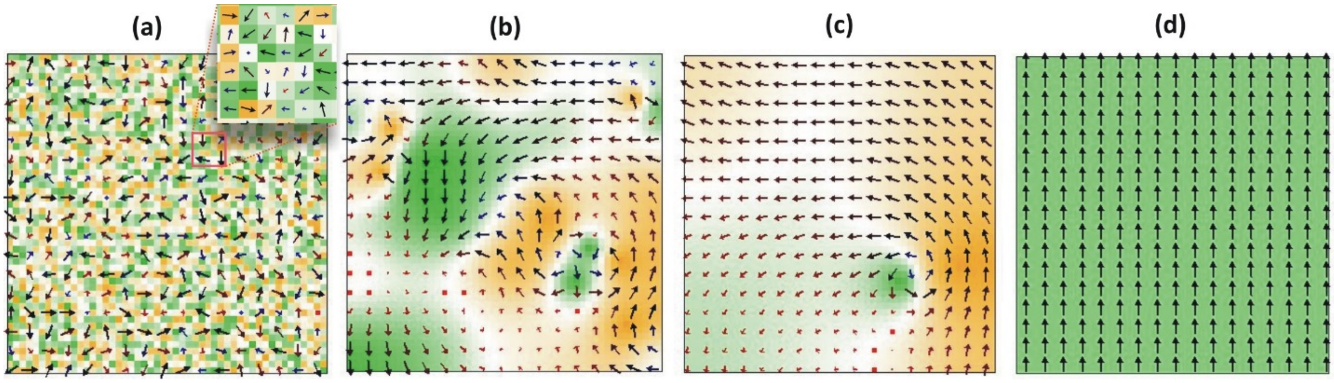

Figure S5. Snapshots of the various simulated state of the Co-doped system. (a) represents the initial ground state (zoomed version is mentioned on top of the image), (b) (c) are intermediate meta-stable states, whereas (d) is a stable energetic ground state of the system.
